# Middle childhood profiles of social-emotional competencies and difficulties differentiate risk of health service presentations with adolescent mental disorders

**DOI:** 10.1177/00048674261426776

**Published:** 2026-03-25

**Authors:** Emma J Carpendale, Melissa J Green, Oliver J Watkeys, Stacy Tzoumakis, Vaughan J Carr, Kristin R Laurens

**Affiliations:** 1 School of Psychology and Counselling, Queensland University of Technology (QUT), Brisbane, QLD, Australia; 2 Centre for Inclusive Education (C4IE), Queensland University of Technology (QUT), Brisbane, QLD, Australia; 3 Centre for Child and Family Studies, Queensland University of Technology (QUT), Brisbane, QLD, Australia; 4 Discipline of Psychiatry and Mental Health, School of Clinical Medicine, University of New South Wales, Sydney, NSW, Australia; 5 School of Criminology and Criminal Justice, Griffith University, Southport, QLD, Australia; 6 Griffith Criminology Institute, Griffith University, Brisbane, QLD, Australia

**Keywords:** Latent profile analysis, mental disorders, dual-factor model of mental health, wellbeing, psychological distress

## Abstract

**Objective:**

The dual-factor model of mental health postulates a role for positive mental health, alongside mental illness, in determining mental health care needs. Informed by this model, the present study delineated profiles of social-emotional competencies and difficulties during middle childhood in a population-based sample of girls and boys and determined their association with adolescent mental disorder diagnoses.

**Method:**

Latent profile analyses were conducted across five indices of social-emotional competency and four indices of psychopathology that were measured by questionnaire self-report among 13,349 girls and 13,488 boys at age ~11 years. The association of the profiles with adolescent presentations to hospital or ambulatory services (ages ~12–17 years) were determined using logistic regression.

**Results:**

Analyses yielded five profiles in each sex: *complete mental health* (44% girls; 42% boys), *average mental health* (30%; 33%), *internalising symptoms with poor relationship skills* (9%; 7%), *externalising symptoms with poor self-management* (12%; 9%) and *low mental health* (5%; 10%). Profiles associated differentially with adolescent presentations with any mental disorder, externalising disorders, internalising disorders and self-harm/suicidal ideation, identified in linked health records. Greater odds of any and specific mental disorder diagnoses were characteristic of all profiles relative to *average mental health* (adjusted odds ratios [aOR]: 1.7–3.3) except *complete mental health* (aOR: 0.7–0.9), with different strengths of association according to profile.

**Conclusion:**

Combining information on social-emotional competencies and psychopathology in middle childhood may help refine the provision of mental health promotion and early intervention to alleviate adolescent mental disorder.

With rates of youth mental disorders and psychological distress increasing globally (Brazel et al., 2023; Sacco et al., 2024; Slade et al., 2024; Tkacz and Brady, 2021), and adolescence marking the peak period of onset for many mental disorders (Solmi et al., 2022), school-based tiered mental health and wellbeing interventions have become a popular, evidence-based avenue of mitigating burden on mental health services (Grové and Laletas, 2020; Humphrey, 2023; Lee et al., 2023). These tiered interventions incorporate universal prevention for all students, alongside targeted interventions for students with emerging psychopathology, and individualised intensive interventions for students with established mental health difficulties. By universally fostering social-emotional wellbeing for all children, these approaches seek to prevent an increasing accumulation of need at the targeted and intensive intervention levels (de Bruin et al., 2023; Greenberg et al., 2017; Guest et al., 2024). They align well with growing evidence in support of a ‘dual-factor model of mental health’, wherein positive mental health and mental illness represent distinct (albeit correlated) factors rather than the extreme ends of a single dimension (Greenspoon and Saklofske, 2001; Suldo and Shaffer, 2008). Positive mental health is defined by the World Health Organisation (2025) as ‘a state of mental well-being that enables people to cope with the stresses of life, realize their abilities, learn and work well, and contribute to their community’. The model postulates that consideration of indicators of social-emotional wellbeing alongside symptoms of ill health may better differentiate children’s need for interventions (Iasiello and Van Agteren, 2020) and help inform the level of intervention required. Informed by the model, the present study sought to examine population patterns in children’s co-presentation of social-emotional competencies and symptoms of psychopathology during middle childhood and determine how these patterns related to children’s subsequent presentations to health services with mental disorders during adolescence.

The dual-factor model of mental health proposes four different expressions (profiles) of social-emotional wellbeing and psychopathology in the population, namely: (1) ‘*complete mental health’*, represented by high wellbeing and low psychopathology; (2) ‘*troubled’*, reflected in low wellbeing and high psychopathology; (3) ‘*vulnerable’*, represented by low wellbeing but also low psychopathology; and (4) ‘*symptomatic but content’*, reflected in high psychopathology but also high wellbeing (Greenspoon and Saklofske, 2001; Lyons et al., 2013). Commonly, prior studies have tested the model by imposing threshold scores to distinguish the four profiles of high versus low levels of wellbeing and psychopathology and then examined their associations with predictors or outcomes (Iasiello and Van Agteren, 2020). More recently, several studies have adopted person-centred techniques as a more flexible and data-driven approach to identifying underlying subgroups within the population, and examined their alignment with the four theorised expressions (Clark et al., 2024). These methods, including latent class/profile analyses (LCA/LPA), classify individuals into homogeneous subgroups based on response patterns across a set of wellbeing and psychopathology indices (Collins and Lanza, 2009).

Varying support for the four expressions in the dual-factor model has emerged from these analyses, conducted in child and adolescent community samples across a diversity of social-emotional wellbeing and psychopathology indicators, as summarised in Table 1. Across these studies, differing numbers of profiles emerged, including 3- (Clark and Malecki, 2022), 4- (Kim et al., 2018; Moore et al., 2019; Petersen et al., 2020) and 5-profile (Gregory et al., 2024) solutions. Multiple investigations were unable to detect a ‘*vulnerable’* profile (Clark and Malecki, 2022; Gregory et al., 2024; Kim et al., 2018; Moore et al., 2019) with average mental health profiles (moderate wellbeing and moderate psychopathology) typically emerging instead (Gregory et al., 2024; Kim et al., 2018; Moore et al., 2019). These divergent findings in the number of profiles and their characteristics may reflect differences in sample size, cultural settings, developmental stages and/or measures used, with some assessments encompassing only internalising or externalising symptoms or only social (interpersonal) or emotional (intrapersonal) wellbeing. Notably, however, LCA/LPA studies stratified by gender have identified consistent profile solutions for girls and boys (Gregory et al., 2024; Kim et al., 2018), but with boys being more likely to present the ‘*complete mental health’* rather than ‘*troubled’* or ‘*symptomatic but content’* profiles (Clark and Malecki, 2022).

**Table 1.** Summary of latent profile/class analyses conducted among children and adolescents and informed by the dual-factor model of mental health.

Study Reference	Location	Sample size	Age at assessment	Indices	Class/Profile Solutions	Equivalent Class/Profile within the Dual-Factor Model
Clark and Malecki (2022)	United States	404	11–14 years (Grades 6–8)	- Life satisfaction (SLSS) - Affect (PANAS-C) - Internalising problems (YSR) - Externalising problems (YSR)	1. Complete mental health (55%) 2. Symptomatic but content (34%) 3. Troubled (11%)	1. Complete mental health 2. Symptomatic but content 3. Troubled
Gregory et al. (2024)	Australia	75,757	8–18 years (Grades 4–12)	- Life satisfaction (SLS) - Optimism (MDI) - Happiness (EPOCH) - Sadness (SPQ) - Worries (WEC)	1. Complete mental health (23%) 2. Good mental health (33%) 3. Moderate mental health (27%) 4. Symptomatic but content (9%) 5. Troubled (8%)	1. Complete mental health — — 2. Symptomatic but content 3. Troubled
Kim et al. (2018)	South Korea	1,757	9–12 years (Grades 4–6)	- Gratitude (SEHS-P) - Zest (SEHS-P) - Optimism (SEHS-P) - Persistence (SEHS-P) - Emotional difficulties (M&Ms) - Behavioural difficulties (M&Ms)	1. Flourishing (14–17%) ^a^ 2. Moderate flourishing (29–38%) 3. Moderate languishing (38–40%) 4. Languishing (8–17%)	1. Complete mental health — — 2. Troubled
Moore et al. (2019)	United States	332	~14–17 years (Grades 9–11)	- Belief in self (SEHS-S) - Belief in others (SEHS-S) - Emotional competence (SEHS-S) - Engaged living (SEHS-S) - Internalising problems (SDQ) - Externalising problems (SDQ)	1. Complete mental health (21–41%)^b^ 2. Moderately mentally healthy (32–44%) 3. Symptomatic but content (20–31%) 4. Troubled (4–6%)	1. Complete mental health — 2. Symptomatic but content 3. Troubled
Petersen et al. (2020)	United Kingdom	3,340	8–9 years (Grades 4–5)	- Subjective Wellbeing (KIDSCREEN-27) - Conduct problems (SDQ) - Emotional symptoms (SDQ)	1. Complete mental health (57%) 2. Vulnerable (13%) 3. Conduct problems but content (12%) 4. Emotional symptoms but content (18%)	1. Complete mental health 2. Vulnerable 3. Symptomatic but content 4. Symptomatic but content

SLSS = Students’ Life Satisfaction Scale (Huebner, 1991); PANAS-C = Positive and negative Affect Scale for Children (Laurent et al., 1999); YSR = Youth Self-Report (Achenbach and Rescorla, 2001); SLS = Satisfaction with Life Scale (Gadermann et al., 2010); MDI = Middle Years Development Index (Schonert-Reichl et al., 2013); EPOCH = EPOCH Measure of Adolescent Wellbeing (Kern et al., 2015); SPQ = Seattle Personality Questionnaire (Kusche et al., 1988); WEC = Wellbeing and Engagement Collection (Gregory et al., 2021); SEHS-*p* = Social Emotional Health Survey – Primary (Furlong et al., 2013); M&Ms = Me and My School Questionnaire (Deighton et al., 2013); SEHS-S = Social-Emotional Health Survey – Secondary (You et al., 2014); SDQ = Strengths and Difficulties Questionnaire (Goodman, 2001); KIDSCREEN-27 (Ravens-Sieberer et al., 2007).

a differentiated among girls and boys.

b differentiated by grade levels.

Limited research has explored longitudinal associations between these childhood/adolescent profiles and later mental illness. In their study of US students (Grades 9–11), Moore et al. (2019) indicated significantly higher later mean depression and anxiety symptoms in Grade 12 for the ‘*troubled’* and ‘*symptomatic but content’* profiles relative to the ‘*complete mental health’* and ‘*moderate mental health’* profiles. Other evidence may be inferred from a study that explored associations between six early childhood profiles (derived on eight teacher-reported indices of social-emotional competencies and difficulties among ~34,000 Canadian Kindergarten students aged ~5 years) and mental health conditions recorded in health records up to the age of 14 years (Thomson et al., 2019). Although not explicitly informed by the dual-factor model, four of the six profiles, were broadly consistent with the ‘*complete mental health*’, ‘*troubled*’ and ‘*vulnerable*’ profiles and a ‘*symptomatic but content’* profile featuring prominent aggressive and hyperactive/inattentive behaviours. In addition, there were two profiles akin to the ‘*moderate’* and ‘*good mental health’* profiles identified by Gregory et al. (2024) and others, though with varied patterns of particular social-emotional competencies and difficulties. After adjusting for various sociodemographic covariates, relative to ‘*complete mental health’*, both the ‘*troubled’* and the ‘*symptomatic but content’* profiles had increased odds of conduct disorders, attention-deficit/hyperactivity disorders (ADHD) and of multiple disorders, but no association with depressive or anxiety disorders. Increased odds of multiple disorders were also apparent for the ‘*vulnerable’* relative to ‘*complete mental health’* profile. Further research is needed to determine whether associations between the profiles and depressive/anxiety disorders might be identified during adolescence, as children mature towards the peak age for onset of these disorders (McGrath et al., 2023; Solmi et al., 2022), and/or when using self-reports gathered during middle childhood relative to kindergarten-teacher reports of children’s social-emotional functioning. Given recognised sex-based disparities in mental health disorder prevalence and patterns (Eaton et al., 2012), research is also needed to determine whether the association of the profiles with later mental disorders varies by sex.

The present study examined these questions using data from a large population-based sample of ~27,000 children drawn from the New South Wales Child Development Study (NSW-CDS; Green et al., 2024). LPA analyses determined middle childhood mental health profiles, separately for girls and boys, according to nine indices (described in Table 2) of social-emotional competencies (*self-awareness, self-management, social awareness, relationship skills* and *responsible decision-making*) and symptoms of psychopathology (*emotional symptoms, peer relationship problems, conduct problems* and *hyperactivity-inattention*). This study subsequently investigated associations between these profiles and later presentations to health services with mental disorder diagnoses during adolescence (~12–17 years). Building on prior Australian research showing girls (cf. boys) at a higher risk of mental health service use, internalising disorders and suicidality (Islam et al., 2020; Watkeys et al., 2024), the study examined, separately by sex, the association of the middle childhood profiles with adolescent health services presentations for any mental disorder, and externalising disorders, internalising disorders and self-harm/suicidal ideation. It was hypothesised that the LPA solutions would demonstrate partial support for the dual-factor model among girls and boys, with the emergence of a ‘*moderate mental health’* profile likely in place of a ‘*vulnerable’* profile. It was also hypothesised that children classified into profiles characterised by lower social-emotional competencies and higher psychopathology scores would demonstrate significantly greater odds of later mental disorders than children in a profile characterised by higher competencies and lower psychopathology scores.

**Table 2.** Social-emotional competencies (from the *Middle Childhood Survey – Social-Emotional Learning*) and social-emotional difficulties (from the *Strengths and Difficulties Questionnaire*).

Domain		Competency	Definition
Social-Emotional competencies	Intrapersonal	Self-Awareness	The ability to understand one’s own emotions, thoughts and behaviours, and have a well-grounded sense of confidence and purpose. Example item: *I easily learn my school work*
		Self-Management	The ability to manage one’s emotions, thoughts and behaviours effectively in different situations and to achieve one’s goals. Example item: *When I am angry, I throw or break things [reversed]*
	Interpersonal	Social Awareness	The ability to understand and empathise with the perspectives of others, including those from diverse backgrounds, cultures and contexts. Example item: *I try to be nice to other people. I care about their feelings*
		Relationship Skills	The ability to establish and maintain healthy and supportive relationships with diverse individuals and groups through effective communication and collaboration. Example item: *My school is a place where I get on well with other students in my class*
	Decision-Making	Responsible Decision-Making	The ability to make caring and constructive decisions about one’s personal behaviour and to consider the consequences of one’s actions. Example item: *I keep my things in order*
Social-Emotional Difficulties	Internalising	Emotional Symptoms	Assesses the experiences of emotional distress, including nervousness, worry and depression. Example item: *I am often unhappy, depressed or tearful*
		Peer Relationship Problems	Assesses difficulties forming positive friendships with peers of the same age and/or experiences of negative peer relationships. Example item: *Other children or young people pick on me or bully me*
	Externalising	Conduct Problems	Assesses behavioural problems, including difficulty controlling temper, disobedience, fighting, lying or stealing. Example item: *I get very angry and often lose my temper*
		Hyperactivity-Inattention	Assesses difficulties with staying still, concentrating, thinking before acting and completing tasks. Example item: *I am easily distracted, I find it difficult to concentrate*

## Method

### Participants and procedure

Participants in this study (*n* *=* 26,837; 49.7% girls) were a population-based subsample of children in the NSW-CDS cohort who completed the self-report Middle Childhood Survey in 2015 (when aged 11–12 years; Laurens et al., 2017) and had linked health records available in the study’s third record linkage (Green et al., 2024). Supplementary Table S1 provides sample demographic details, relative to the full cohort. As at March 2021 (the last date of linked mental health records), the study sample was, on average, 17.52 years old (SD = 0.38; Median = 17.50; IQR = 17.25–17.75 years). Children who had mental disorder presentations prior to January 2016 (*n* = 1,903) were retained in the derivation of mental health profiles but excluded from analyses of the association of profiles with adolescent mental disorder diagnoses.

Ethical approval (HREC/18/CIPHS/49) for the linkage was provided by the NSW Population and Health Services (2019/ETH01571) and ACT Health Human Research Ethics Committees (2019.STE.00184), with relevant data custodian approvals. Probabilistic linkage of records was conducted by the Centre for Health and Record Linkage, managed by a third party under a waiver of consent process consistent with the Australian National Health and Medical Research Council’s (2007) National Statement of Ethical Conduct in Human Research. False linkage rate across all record sets was estimated at 0.5%.

### Measures

#### Social-emotional competencies and difficulties

Children’s self-reports of five social-emotional competencies (as defined by the Collaborative for Academic, Social and Emotional Learning [CASEL], 2005, 2025) were assessed using the *Middle Childhood Survey–Social-Emotional Learning* (MCS-SEL; Carpendale et al., 2023a), namely: self-awareness (four items), self-management (three items), social awareness (five items), relationship skills (five items) and responsible decision-making (three items). Internalising and externalising psychopathology were assessed via the four self-report scales (five items per scale) of the *Strengths and Difficulties Questionnaire* (SDQ; Goodman, 2001), namely: emotional symptoms, peer relationship problems, conduct problems and hyperactivity/inattention. All items were rated on a three-level response scale: *not true* (scored 0), *somewhat true* (1) and *certainly true* (2). Internal consistency of the five competencies in this sample is satisfactory (McDonald’s ω: 0.65–0.76; Carpendale et al., 2023a; 2023b) and acceptable to good for the four psychopathologies (ordinal α: 0.69–0.80; Laurens et al., 2017). LPA was conducted on averaged, centred scores (according to sex-based means) for these nine constructs.

#### Mental disorders

Health service contacts for mental disorder diagnoses were recorded in any of four record sets provided by the NSW Ministry of Health: *Admitted Patient Data Collection* (APDC; records from January 2002–March 2021; including presentations in public hospitals, public psychiatric hospitals, multi-purpose services, private hospitals and private day procedure centres in NSW); *Emergency Department Data Collection* (EDDC; January 2005–March 2021; including presentations at emergency departments of public hospitals and in-scope contracted private hospitals in NSW); *NSW Mental Health Ambulatory data collection* (MH-AMB; November 2002–December 2020, which includes care provided to non-admitted patients in mental health day programmes, psychiatric outpatient and community outreach services [e.g. home visits]); and *Controlled Drugs Data Collection* (CoDDaC: June 2004–June 2020, which includes the prescription of Schedule 8 drugs such as psychostimulants).

Four binary outcomes were explored: (1) any mental disorder diagnoses; (2) externalising disorders, including conduct disorder, mixed disorders of conduct and emotion, cluster B personality disorders and substance use disorders; (3) internalising disorders, including affective and anxiety disorders and (4) self-harm or suicidal ideation presentations (Supplementary Table S2 details the ICD-10-AM codes related to these outcomes). Presentation of these disorders from January 2016 (the year after Middle Childhood Survey completion in 2015) were coded 1. The reference group (coded 0) for all analyses was the group of children who had no recorded mental health diagnoses at any time.

#### Sociodemographic variables

Each child’s sex (as assigned at birth) and Aboriginal and Torres Strait Islander background (0 = non-Indigenous; 1 = Indigenous) was determined by consensus across NSW-CDS linked records. Area socioeconomic disadvantage was coded from the child’s residential postcode in 2015 (0 = bottom four quintiles, reflecting less disadvantage; 1 = highest quintile, reflecting greatest disadvantage), according to the Socio-Economic Indexes for Areas (SEIFA) Index of Relative Socio-Economic Disadvantage (IRSD) ratings (Australian Bureau of Statistics, 2011). Children’s geographical remoteness was coded according to children’s residential postcodes using ratings from the Accessibility and Remoteness Index of Australia (0 = major cities; 1 = inner regional, outer regional, remote and very remote areas; Department of Health and Aged Care, 2001).

### Statistical analysis

#### Latent profile analyses

Latent profiles were specified in Mplus version 8 (Muthén and Muthén, 1998) for girls and boys, separately. Models were repeated, specifying two to *k* profiles, until the model’s best loglikelihood value was not replicated, indicating a local solution rather than a general solution. Profiles were estimated using Maximum Likelihood with Robust standard errors, accounting for non-normality (Vermunt and Magidson, 2002). Models were specified with 500 initial-stage random starts, 50 final-stage optimizations (Nylund-Gibson and Choi, 2018) and, for the time-intensive likelihood bootstrap tests, 100 draws were requested (Tein et al., 2013). The optimal model (i.e. number of profiles) was determined by evaluating class separation (success of classification of individuals) via entropy values; model parsimony via log-likelihood values, Akaike Information Criteria (AIC) and Bayesian Information Criterion (BIC); and distributional properties via the adjusted Lo-Mendell-Rubin adjusted likelihood ratio test (LMR-LRT) and bootstrapped likelihood ratio test (BLRT) (Weller et al., 2020). Entropy values of 0.40, 0.60 and 0.80 were interpreted as low, moderate and high class separation, respectively (Clark and Muthén, 2009). Smaller loglikelihood, AIC and BIC values were optimal, and significant (*p* < 0.05) LMR-LRT and BLRT values indicated improved model fit relative to the previous solution. Solutions with profile sizes of ⩾5% were preferred, on account of the practical applications of small profiles (Weller et al., 2020). Profile enumeration also considered practical and theoretical relevance.

#### Profiles and later mental health service use

Once profile solutions for girls and boys were selected and the most likely profile memberships extracted for each child, a series of bivariate (unadjusted) logistic regressions were conducted to explore associations between the mental health profiles (relative to an average/moderate mental health profile) and mental health presentations, with children who had no presentations as the reference group. Analyses were repeated controlling for sociodemographic covariates (Aboriginal/Torres Strait Islander background, area-based socioeconomic disadvantage and geographical remoteness). The logistic regression analyses yielded odds ratios and their 95% confidence intervals (CIs), with significance denoted by CIs that did not cross 1.00. Odds ratios of 1.00–1.49 (or 1.00–0.67) were interpreted as small effects, 1.50–2.49 (or 0.66–0.40) as medium, 2.50–4.00 (or 0.39–0.25) as large and >4.00 (or <0.25) as very large effects (Rosenthal, 1996).

## Results

### Latent profile analysis

For girls and boys, solutions are reported up to nine profiles (Table 3), beyond which the LMR-LRT was consistently non-significant, indicating no improvement in fit. With each increase in the number of profiles specified, small improvements were observed in AIC and BIC. For both samples, LMR-LRT was non-significant at the six-profile solution, indicating six profiles did not significantly improve fit above the five-profile model. In both samples, a five-profile solution was the last model before the smallest profiles consistently comprised less than 5% of children and before posterior probabilities fell below 80%. Accordingly, the most likely profile memberships from the five-profile solutions for girls and boys were extracted for each child.

**Table 3.** Model fit comparison of latent profile analysis of social-emotional competencies and psychopathology among girls and boys.

Number of Profiles	Log Likelihood	AIC	BIC	Entropy	LMR-LRT	BLRT	Lowest Class Probability	Lowest Class Size	Lowest Class Proportion
**Girls**									
1	-65,287.92	130,611.84	130,746.83	-	-	-	-	-	-
2	-52,519.67	105,095.33	105,305.31	0.868	25,270.48***	25,536.51***	0.94	3,674	27.5
3	-48,632.03	97,340.07	97,625.04	0.828	7,694.27***	7,775.27***	0.89	1,470	11.0
4	-47,429.13	94,954.27	95,314.23	0.808	2,380.74***	2,405.80***	0.85	603	4.5
5	-46,197.51	92,511.03	92,945.98	0.806	2,437.59***	2,463.24***	0.82	666	5.0
6	-45,344.50	90,825.00	91,334.95	0.820	1,688.25	1,706.03***	0.80	506	3.8
7	-44,689.24	89,534.49	90,119.42	0.795	1,296.86*	1,310.51***	0.77	486	3.6
8	-44,105.34	88,386.67	89,046.60	0.811	1,155.65	1,167.81***	0.77	389	2.9
9	-43,624.64	87,445.29	88,180.21	0.803	951.37	961.38***	0.74	264	2.0
**Boys**									
1	-71,714.42	143,464.84	143,600.01	-	-	-	-	-	-
2	-60,098.68	120,253.36	120,463.63	0.829	22,989.72***	23,231.48***	0.93	4,583	34.0
3	-56,847.44	113,770.89	114,056.25	0.795	6,434.81***	6,502.48***	0.88	2,024	15.0
4	-55,765.63	111,627.27	111,987.73	0.798	2,141.10*	2,163.62***	0.81	1,363	10.1
5	-54,733.85	109,583.71	110,019.26	0.795	2,042.09**	2,063.56***	0.80	936	6.9
6	-53,920.18	107,976.37	108,487.02	0.787	1,610.41	1,627.34***	0.80	600	4.4
7	-53,240.04	106,636.07	107,221.82	0.780	1,346.14	1,360.30***	0.76	452	3.4
8	-52,619.87	105,415.73	106,076.58	0.796	1,227.43	1,240.34***	0.76	327	2.4
9	-52,198.94	104,593.88	105,329.81	0.759	833.10*	841.86***	0.71	259	1.9

AIC = Akaike Information Criterion; BIC = Bayesian Information Criterion; LMR-LRT = Lo-Mendell-Rubin Adjusted Likelihood Ratio Test; BLRT = Bootstrapped Likelihood Ratio Test.

*** *p* < .001; **p < .010; *p < .050

**Figure 1.**
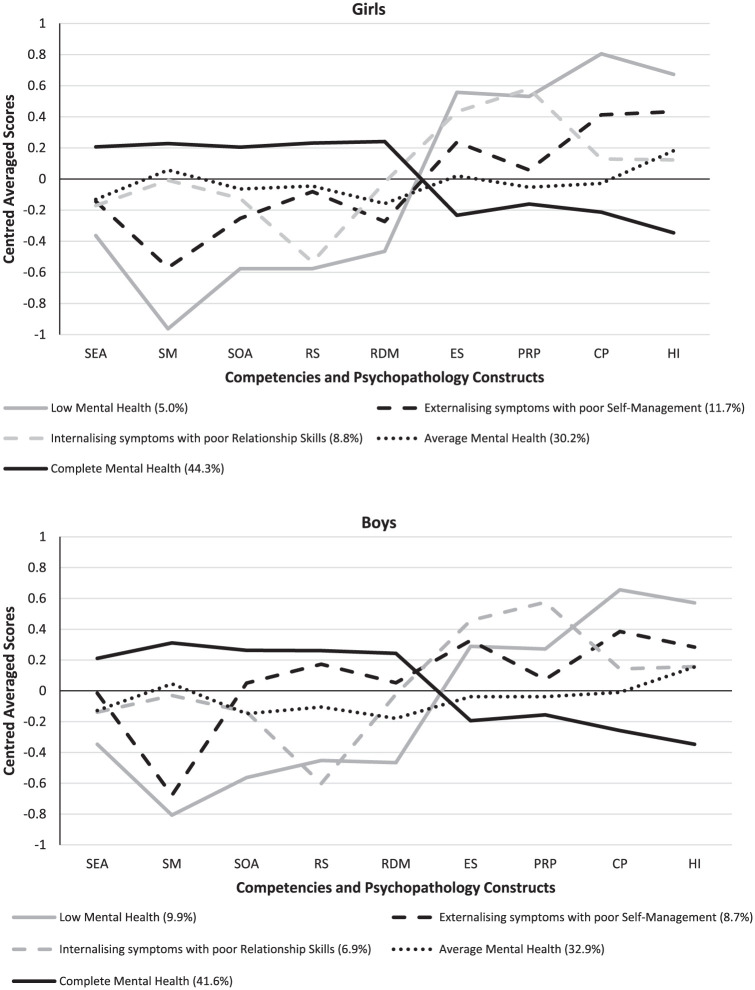
Latent profile analysis solutions evidencing five profiles for girls and boys. SEA = Self-Awareness; SM = Self-Management; SOA = Social Awareness; RS = Relationship Skills; RDM = Responsible Decision-Making; ES = Emotional Symptoms; PRP = Peer Relationship Problems; CP = Conduct Problems; HI = Hyperactivity/Inattention.

Mean values of the nine constructs for children in each of the five profiles are displayed in Figure 1 (and detailed in Supplementary Table S3). Profile patterns were sufficiently comparable for girls and boys to support consistent naming, albeit cross-tabulation analyses revealed small but significantly different prevalence distributions by sex for each of the five profiles. Prevalences for girls and boys in each profile were: *complete mental health* (*complete-MH*: 44.3% girls; 41.6% boys); *average mental health* (*average-MH*: 30.2%; 32.9%); *externalising symptoms with poor self-management* (*externalising-SM*: 11.7%; 8.7%); *internalising symptoms with poor relationship skills* (*internalising-RS*: 8.8%; 6.9%); and *low mental health* (*low-MH*: 5.0%; 9.9%). Despite the overall similarities in profile patterns between girls and boys, some distinctions were noteworthy. Among children in the *externalising-SM* profile, boys had above-average relationship skills whereas girls reported average relationship skills. Girls in the *low-MH* profile demonstrated generalised psychopathology, with the highest scores on internalising and externalising symptoms of any profile, whereas boys in this profile demonstrated lower levels of internalising symptoms than boys in the *internalising-RS* profile, and were instead characterised by pronounced externalising symptoms.

### Profiles’ associations with adolescent mental health service presentations

Table 4 presents the distribution of children within each profile according to adolescent health services presentations with mental disorder diagnoses (aged ~12–17 years). Table 5 displays the odds of children in each profile presenting with mental disorders, each relative to *average-MH*. For the most part, children from the *low-MH*, *externalising-SM* and *internalising-RS* profiles had significantly greater odds of mental disorders across all four outcomes. After adjusting for sociodemographic covariates, children in these three risk profiles were 1.7 to 3.3 times as likely to have any mental disorder in adolescence relative to *average-MH*, representing moderate to large effects. For the most part, children with *low-MH* demonstrated the largest odds of specific mental disorders in adolescence (adjusted odds ratios [aOR]: girls = 3.4–4.9, boys = 1.9–3.3). Children in the *externalising-SM* and *internalising-RS* profiles demonstrated elevated odds of all outcomes, with *externalising-SM* having greatest odds of externalising disorders (aOR: girls = 2.5, boys = 2.2) and *internalising-RS* having greatest odds of internalising disorders (aOR: girls = 2.4, boys = 2.0) and self-harm/suicidal ideation (aOR: girls = 2.6, boys = 1.9). No significant difference was found in the odds of self-harm/suicidal ideation between boys in the *externalising-SM* and *average-MH* profiles. Children with *complete-MH* had significantly reduced odds of all four outcomes relative to *average-MH* (aOR = 0.4–0.9), with two exceptions: for girls, no significant differences between *complete-MH* and *average-MH* were observed in odds of externalising disorders nor self-harm/suicidal ideation.

**Table 4.** Mental health presentations post-2015 (ages ~12–17 years) according to mental health profiles in middle childhood.

	Sample proportion		No presentations		Any mental health presentation		Externalising disorders		Internalising disorders		Self-Harm/Suicidal Ideation	
	%	(n)	%	(n)	%	(n)	%	(n)	%	(n)	%	(n)
**Girls**												
Total Sample (n=13,349)			84.9	(11,330)	10.3	(1,371)	1.7	(228)	5.8	(770)	3.4	(456)
Low Mental Health	5.0	(666)	65.7	(431)	23.9	(159)	4.1	(27)	14.9	(99)	10.7	(71)
Externalising symptoms with poor Self-Management	11.7	(1,565)	78.3	(1,225)	14.5	(227)	3.5	(54)	8.6	(135)	5.4	(85)
Internalising symptoms with poor Relationship Skills	8.8	(1,171)	76.0	(890)	16.7	(196)	2.1	(25)	10.3	(121)	6.1	(71)
Average Mental Health	30.2	(4,037)	86.5	(3,492)	8.7	(352)	1.4	(58)	4.8	(193)	2.6	(105)
Complete Mental Health	44.3	(5,910)	89.5	(5,292)	7.4	(437)	1.1	(64)	3.8	(222)	2.1	(124)
**Boys**												
Total Sample (n=13﻿,488)			84.8	(11,433)	5.9	(800)	1.3	(148)	2.6	(344)	1.2	(163)
Low Mental Health	9.9	(1,336)	70.5	(942)	10.3	(138)	2.6	(35)	4.0	(54)	2.6	(35)
Externalising symptoms with poor Self-Management	8.7	(1,172)	77.6	(910)	10.0	(116)	2.0	(23)	4.2	(49)	1.5	(18)
Internalising symptoms with poor Relationship Skills	6.9	(936)	78.2	(732)	8.4	(79)	1.8	(17)	4.5	(42)	2.1	(20)
Average Mental Health	32.9	(4,436)	85.4	(3,790)	5.6	(250)	0.9	(41)	2.5	(113)	1.3	(57)
Complete Mental Health	41.6	( 5,608)	90.2	(5,059)	3.9	(217)	0.6	(32)	1.5	(86)	0.6	(33)

Excludes children with mental health service presentations prior to 01-Jan-2016 (*n* = 1,903).

**Table 5.** Logistic regression results for mental health presentations according to mental health profile.

		Any mental presentation	Externalising disorders	Internalising disorders	Self-harm/Suicidal ideation
**Girls**					
Low Mental Health	uOR [95% CIs]	**3.66 [2.96, 4.53]**	**3.77 [2.36, 6.02]**	**4.16 [3.20, 5.40]**	**5.48 [3.99, 7.53]**
	aOR [95% CIs]	**3.26 [2.63, 4.05]**	**3.36 [2.09, 5.39]**	**3.60 [2.75, 4.70]**	**4.91 [3.56, 6.78]**
Externalising symptoms with poor Self-Management	uOR [95% CIs]	**1.84 [1.54, 2.20]**	**2.65 [1.82, 3.87]**	**1.99 [1.59, 2.51]**	**2.31 [1.72, 3.09]**
	aOR [95% CIs]	**1.72 [1.44, 2.07]**	**2.46 [1.69, 3.60]**	**1.83 [1.45, 2.31]**	**2.15 [1.60, 2.88]**
Internalising symptoms with poor Relationship Skills	uOR [95% CIs]	**2.19 [1.81, 2.64]**	**1.69 [1.05, 2.72]**	**2.46 [1.94, 3.12]**	**2.65 [1.95, 3.62]**
	aOR [95% CIs]	**2.11 [1.74, 2.55]**	**1.63 [1.01, 2.63]**	**2.36 [1.86, 3.01]**	**2.56 [1.87, 3.50]**
Average Mental Health*					
Complete Mental Health	uOR [95% CIs]	**0.82 [0.71, 0.95]**	0.73 [0.51, 1.04]	**0.76 [0.62, 0.93]**	0.78 [0.60, 1.01]
	aOR [95% CIs]	**0.85 [0.73, 0.98]**	0.75 [0.52, 1.07]	**0.79 [0.64, 0.96]**	0.80 [0.62, 1.04]
**Boys**					
Low Mental Health	uOR [95% CIs]	**2.22 [1.78, 2.77]**	**3.44 [2.18, 5.42]**	**1.92 [1.38, 2.68]**	**2.47 [1.61, 3.79]**
	aOR [95% CIs]	**2.13 [1.70, 2.66]**	**3.26 [2.06, 5.16]**	**1.89 [1.35, 2.64]**	**2.46 [1.60, 3.78]**
Externalising symptoms with poor Self-Management	uOR [95% CIs]	**1.93 [1.53, 2.44]**	**2.34 [1.40, 3.91]**	**1.81 [1.28, 2.55]**	1.32 [0.77, 2.25]
	aOR [95% CIs]	**1.87 [1.48, 2.37]**	**2.20 [1.31, 3.70]**	**1.78 [1.26, 2.51]**	1.31 [0.77, 2.24]
Internalising symptoms with poor Relationship Skills	uOR [95% CIs]	**1.64 [1.26, 2.13]**	**2.15 [1.21, 3.80]**	**1.92 [1.34, 2.77]**	**1.82 [1.09, 3.04]**
	aOR [95% CIs]	**1.66 [1.27, 2.17]**	**2.18 [1.23, 3.86]**	**1.96 [1.36, 2.82]**	**1.85 [1.10, 3.09]**
Average Mental Health*					
Complete Mental Health	uOR [95% CIs]	**0.65 [0.54, 0.78]**	**0.59 [0.37, 0.93]**	**0.57 [0.43, 0.76]**	**0.43 [0.28, 0.67]**
	aOR [95% CIs]	**0.68 [0.56, 0.82]**	**0.61 [0.38, 0.97]**	**0.59 [0.44, 0.78]**	**0.44 [0.29, 0.68]**

* Reference group; Bolded text = *p* < .05; non-bolded text = *p* > .05; CI = confidence intervals; uOR = unadjusted odds ratios; aOR = adjusted odds ratios accounting for Aboriginal and Torres Strait Islander background (No [coded 0] and Yes [1]), Socio-Economic Indexes for Areas Index of Relative Socio-Economic Disadvantage quintile (bottom quintiles of disadvantage [0], top quintile of disadvantage [1]), and Accessibility and Remoteness Index of Australia geographical region (major cities [0], inner and outer regional, remote, or very remote [1]).

## Discussion

Latent profile analyses of middle childhood (age ~11 years) social-emotional competencies and difficulties yielded a five-profile solution for girls and boys, and these profiles differentially related to health service presentations for mental disorders during adolescence (ages ~12–17 years). Between 42–44% of girls and boys were classified as demonstrating *complete mental health* (high competencies, low psychopathology). Another 30–33% of children demonstrated *average mental health*, while the remaining 25–26% of children demonstrated profiles of *low mental health* (girls = 5%, boys = 10%), *externalising symptoms with poor Self-Management* (girls = 12%, boys = 9%) or *internalising symptoms with poor Relationship Skills* (girls = 9%, boys = 7%). Children in these symptomatic and low mental health profiles demonstrated between a 1.7- to 3.3-times greater odds of mental disorders across the adolescent period, relative to *average-MH*. That is, these differentiated profiles of competencies and difficulties distinguished children at risk of presentations with any, and with particular, adolescent mental disorder diagnoses, providing information that may help the tailoring of tiered interventions for children according to their co-presentation of mental health and illness.

This comprehensive exploration of patterns of middle childhood interpersonal and intrapersonal competencies and internalising and externalising psychopathology identified an *average/moderate mental health* profile rather than a ‘*vulnerable’* profile (low psychopathology, low wellbeing), as reported by others (Gregory et al., 2024; Kim et al., 2018; Moore et al., 2019). Similarly to the prior study that assessed both internalising and externalising psychopathology (Petersen et al., 2020), two distinct profiles emerged separating children according to their experiences of these symptoms. These *externalising-SM* and *internalising-RS* profiles align somewhat with the theoretically proposed ‘*symptomatic but content’* class, defined by high psychopathology symptoms and high wellbeing (Greenspoon and Saklofske, 2001; Lyons et al., 2013). Excepting self-management, children in the *externalising-SM* profile demonstrated average (girls) to above average (boys) levels of all other social-emotional competencies. Similarly, children in the *internalising-RS* profile reported average levels of all competencies, excepting relationship skills. These profiles indicate that social-emotional competence can exist despite psychopathology symptoms, excepting perhaps selected competencies closely aligned with their respective indicators of difficulties (i.e. self-management with conduct problems, and relationships skills with peer relationship problems). These competencies denote additional protective factors that might be bolstered to mitigate against psychopathology symptoms. School-based promotion of these skills requires the provision of universal social-emotional learning programmes targeting all five competencies (Carpendale et al., 2025), but where indicated, this whole-school provision should be supplemented by targeted and intensive interventions delivered to students requiring additional support, such as via small group-based activities or individualised support plans delivered to children at risk of or experiencing mental health difficulties (Close et al., 2023). Tailoring of these higher-tier interventions should be informed by comprehensive assessment of a student’s social-emotional competencies alongside their difficulties, so that they respond to specific needs (e.g. relationships skills promotion for *internalising-RS* children).

This study demonstrated longitudinal associations between middle childhood mental health profiles and adolescent mental disorders. The *complete-MH* profile was, for the most part, protective against later disorders relative to *average-MH*. In a prior study (Moore et al., 2019), ‘*symptomatic but content’* and ‘*troubled’* profiles were later characterised by greater depression and anxiety symptoms than ‘*moderate’* and ‘*complete mental health’* profiles. Children classified here into the symptomatic (*externalising-SM; internalising-RS*) or *low-MH* profiles similarly had significantly greater odds of any, internalising, and externalising disorder presentations, relative to the *average-MH* profile, even when adjusting for sociodemographic covariates. Findings are also consistent with research indicating homotypic *and* heterotypic continuity between internalising and externalising disorders (Copeland et al., 2013; Shevlin et al., 2017). That is, children with pronounced externalising symptoms (*low-MH; externalising-SM*) and internalising symptoms (*low-MH; internalising-RS*) demonstrated significantly greater odds (cf. *average-MH*) of both externalising and internalising disorders. Whereas odds of self-harm/suicidal ideation among girls were significantly higher for all three risk profiles relative to *average-MH*, this was not replicated among boys in the *externalising-SM* profile. This may reflect the paradox by which males are less likely to engage in self-harm than females (Diggins et al., 2024), despite being more likely to die by suicide (Barrigon and Cegla-Schvartzman, 2020). Alternatively, the greater relationship skills of *externalising-SM* boys, relative to girls in the equivalent profile, may provide the foundation for stronger relationships with family and peers, and act as a protective factor.

A strength of this study was the use of a large, representative sample of Australian young people followed over time, but several limitations are notable. Non-significant differences in odds of mental disorders could be a result of the small number of individuals with mental disorders among some of the profiles (e.g. 1.5% of *externalising-SM* boys with self-harm/suicidal ideation presentations and 1.1% of *complete-MH* girls with externalising disorders), resulting in wide confidence intervals that broach 1.0 (Reeve, 2018). Data was limited to hospital and ambulatory service presentations for mental disorders and does not account for presentations to other types of health services, such as general practitioners, private psychologists or psychiatrists or counsellors. Children who had diagnoses recorded prior to/in the year of the Middle Childhood Survey (2015; Laurens et al., 2017) were removed from logistic regression analyses (*n* = 1,903), as we sought to explore the association of middle childhood profiles with *later* mental health service use among children who had not previously accessed such services. Thus, this study was not equipped to explore associations between these profiles and neurodevelopmental disorders, for which peak onset is in early childhood (Solmi et al., 2022). Finally, this study employed brief measures of social-emotional competencies and difficulties (3–5 items per scale), limiting coverage of these domains. Replication using more comprehensive measures would ascertain the consistency of profile solutions.

Future longitudinal research could explore potential predictors of membership in each of the profiles, such as early childhood mental disorders, parental mental illness or adverse childhood experiences. Continued follow-up of the sample into early adulthood would capture more mental health presentations, particularly for disorders that peak in onset during adulthood (e.g. substance use, mood and personality disorders and schizophrenia; Solmi et al., 2022). Where this study was limited to self-report assessment of children’s perceived social-emotional competencies and difficulties at age ~11 years, future research could determine whether these profiles are replicated among different age groups and using teacher- and/or parent-report data.

This study demonstrates the utility of middle childhood mental health profiles that account for both social-emotional competencies and difficulties in indexing risk for mental disorders in adolescence (ages ~12–17 years). Middle childhood represents an opportune time for prevention and early intervention, prior to the difficult transition to adolescence – the peak period of onset for many mental disorders (McGrath et al., 2023; Solmi et al., 2022). Social-emotional competencies are teachable and can be promoted in schools via effective, evidence-based programming (Carpendale et al., 2025; Durlak et al., 2022). Further research is required to determine the effectiveness of these programmes for averting mental health difficulties and to inform where the provision of targeted and intensive supports – tailored to students’ social-emotional needs and emerging/established psychopathology – may be required in addition to the whole-school teaching of all competencies.

## Supplemental Material

sj-docx-1-anp-10.1177_00048674261426776 – Supplemental material for Middle childhood profiles of social-emotional competencies and difficulties differentiate risk of health service presentations with adolescent mental disorders Supplemental material, sj-docx-1-anp-10.1177_00048674261426776 for Middle childhood profiles of social-emotional competencies and difficulties differentiate risk of health service presentations with adolescent mental disorders by Emma J Carpendale, Melissa J Green, Oliver J Watkeys, Stacy Tzoumakis, Vaughan J Carr and Kristin R Laurens in Australian & New Zealand Journal of Psychiatry
