# Supplementary material for: Middle childhood profiles of social-emotional competencies and difficulties differentiate risk of health service presentations with adolescent mental disorders
Source: Aust N Z J Psychiatry. 2026 Mar 25;60(7):653–65. doi: 10.1177/00048674261426776 (PMC13291414; doi:10.1177/00048674261426776)
Supplement: sj-docx-1-anp-10.1177_00048674261426776 – Supplemental material for Middle childhood profiles of social-emotional competencies and difficulties differentiate risk of health service presentations with adolescent mental disorders [file sj-docx-1-anp-10.1177_00048674261426776.docx]

**Middle Childhood Profiles of Social-Emotional Competencies and Difficulties Differentiate Risk of Health Service Presentations with Adolescent Mental Disorders**

***Supplementary Materials***

**Contents:**

*Supplementary Table S1:* Sociodemographic characteristics of the sample, relative to the full NSW-CDS cohort. 2

*Supplementary Table S2:* Details of mental disorder categories examined. 2

*Supplementary Table S3.* Mean social-emotional competency and difficulty scores for the 5-profile solution. 5

**Supplementary Table S1.** Sociodemographic characteristics of the sample, relative to the full NSW-CDS cohort.

|  | NSW-CDS Cohort  (*n* = 91,597) | Study Sample  (*n* = 26,837) | Girls  (*n* = 13,349) | Boys  (*n* = 13,488) |
| --- | --- | --- | --- | --- |
| Aboriginal or Torres Strait Islander | 7,970 (8.7%) | 2,201 (8.2%) | 1,103 (8.3%) | 1,098 (8.1%) |
| Socioeconomic Disadvantage (SEIFA IRSD) |  |  |  |  |
| Quintile 2-5 (least disadvantage) | 61,571 (80.6%^a^) | 21,864 (81.5%) | 10,844 (81.2%) | 11,020 (81.7%) |
| Quintile 1 (most disadvantage) | 14,778 (19.4%^a^) | 4,973 (18.5%) | 2,505 (18.8%) | 2,468 (18.3%) |
| Geographical region (ARIA) |  |  |  |  |
| Major cities | 58,988 (77.3%^b^) | 19,312 (72.0%) | 9,619 (72.1%) | 9,693 (71.9%) |
| Regional/Remote ^a^ | 17,360 (22.7%^b^) | 7,525 (28.0%) | 3,730 (27.9%) | 3,795 (28.1%) |

*Note.* ^a^ includes outer regional, inner regional, remote, and very remote regions; SEIFA IRSD = Socio-economic Indexes for Area Index of Relative Socio-economic Disadvantage; ARIA = Accessibility/Remoteness Index of Australia.

**Supplementary Table S2.** Details of mental disorder categories examined.

| **Disorder Categories** | **ICD-10-AM Codes** |
| --- | --- |
| **Externalising Disorders** | |
| Conduct disorders | F91.0 – Conduct disorder confined to the family context |
|  | F91.1 – Unsocialized conduct disorder |
|  | F91.2 – Socialized conduct disorder |
|  | F91.3 – Oppositional defiant disorder |
|  | F91.8 – Other conduct disorders |
|  | F91.9 – Conduct disorder, unspecified |
| Mixed disorders of conduct and emotions | F92.0 – Depressive conduct disorder |
|  | F92.8 – Other mixed disorders of conduct and emotions |
|  | F92.9 – Mixed disorder of conduct and emotions, unspecified |
| Cluster B personality disorders | F60.2 – Dissocial personality disorder |
|  | F60.3 – Emotionally unstable personality disorder |
|  | F60.4 – Histrionic personality disorder |
| Substance use disorders |  |
| Diagnosed substance use disorder | F1.0 – Acute intoxication |
|  | F1.1 – Harmful use |
|  | F1.2 – Dependence syndrome |
|  | F1.3 – Withdrawal state |
|  | F1.4 – Withdrawal state with delirium |
|  | F1.6 – Amnesic syndrome |
|  | F1.8 – Other mental and behavioural disorders |
|  | F1.9 – Unspecified mental and behavioural disorders |
| Opioid treatment program (CoDDaC) | Any opioid treatment record from the Opioid Treatment Program dataset. |
| **Internalising Disorders** | |
| Affective disorders |  |
| Depressive episode | F32.0 – Mild depressive episode |
|  | F32.1 – Moderate depressive episode |
|  | F32.2 – Severe depressive episode without psychotic symptoms |
|  | F32.3 – Severe depressive episode with psychotic symptoms |
|  | F32.8 – Other depressive episodes |
|  | F32.9 – Depressive episode, unspecified |
| Recurrent depressive disorder | F33.0 – Recurrent depressive disorder, current episode mild |
|  | F33.1 – Recurrent depressive disorder, current episode moderate |
|  | F33.2 – Recurrent depressive disorder, current episode severe without psychotic symptoms |
|  | F33.3 – Recurrent depressive disorder, current episode severe with psychotic symptoms |
|  | F33.4 – Recurrent depressive disorder, currently in remission |
|  | F33.8 – Other recurrent depressive disorders |
|  | F33.9 – Recurrent depressive disorder, unspecified |
| Persistent mood [affective] disorders | F34.0 – Cyclothymia |
|  | F34.1 – Dysthymia |
|  | F34.8 – Other persistent mood [affective] disorders |
|  | F34.9 – Persistent mood [affective] disorder, unspecified |
| Other mood [affective] disorders | F38.0 – Other single mood [affective] disorders |
|  | F38.1 – Other recurrent mood [affective] disorders |
|  | F38.8 – Other specified mood [affective] disorders |
| Unspecified mood [affective] disorders | F39.0 – Unspecified mood [affective] disorders |
| Postnatal depression | F53.0 – Mild mental and behavioural disorders associated with the puerperium, not elsewhere classified |
|  | F53.1 – Severe mental and behavioural disorders associated with the puerperium, not elsewhere classified |
|  | F53.8 – Other mental and behavioural disorders associated with the puerperium, not elsewhere classified |
|  | F53.9 – Puerperal mental disorder, unspecified |
| Anxiety disorders |  |
| Dissociative/conversion disorders | F44.0 – Dissociative amnesia |
|  | F44.1 – Dissociative fugue |
|  | F44.2 – Dissociative stupor |
|  | F44.3 – Trance and possession disorders |
|  | F44.4 – Dissociative motor disorders |
|  | F44.5 – Dissociative convulsions |
|  | F44.6 – Dissociative anaesthesia and sensory loss |
|  | F44.7 – Mixed dissociative [conversion] disorders |
|  | F44.8 – Other dissociative [conversion] disorders |
|  | F44.9 – Dissociative [conversion] disorder, unspecified |
| Obsessive compulsive disorders | F42.0 – Predominantly obsessional thoughts or ruminations |
|  | F42.1 – Predominantly compulsive acts [obsessional rituals] |
|  | F42.2 – Mixed obsessional thoughts and acts |
|  | F42.8 – Other obsessive-compulsive disorders |
|  | F42.9 – Obsessive-compulsive disorder, unspecified |
| Phobic anxiety disorders | F40.0 - Agoraphobia |
|  | F40.1 – Social phobias |
|  | F40.2 – Specific (isolated) phobias |
|  | F40.8 – Other phobic anxiety disorders |
|  | F40.9 – Phobic anxiety disorder, unspecified |
| Other anxiety disorders | F41.0 – Panic disorder [episodic paroxysmal anxiety] |
|  | F41.1 – Generalized anxiety disorder |
|  | F41.2 – Mixed anxiety and depressive disorder |
|  | F41.3 – Other mixed anxiety disorder |
|  | F41.8 – Other specified anxiety disorders |
|  | F41.9 – Anxiety disorder, unspecified |
| Emotional disorders with onset specific to childhood | F93.0 – Separation anxiety disorders of childhood |
|  | F93.1 – Phobic anxiety disorder of childhood |
|  | F93.2 – Social anxiety disorder of childhood |
|  | F93.3 – Sibling rivalry disorder |
|  | F93.8 – Other childhood emotional disorders |
|  | F93.9 – Childhood emotional disorder, unspecified |
| Somatoform disorders | F45.0 – Somatization disorder |
|  | F45.1 – Undifferentiated somatoform disorder |
|  | F45.2 – Hypochondriacal disorder |
|  | F45.3 – Somatoform autonomic dysfunction |
|  | F45.4 – Persistent somatoform pain disorder |
|  | F45.8 – Other somatoform disorders |
|  | F45.9 – Somatoform disorder, unspecified |
| Other neurotic disorders | F48.0 – Neurasthenia |
|  | F48.1 – Depersonalization – derealization syndrome |
|  | F48.8 – Other specified neurotic disorders |
|  | F48.9 – Neurotic disorder, unspecified |
| Stress reactions | F43.0 – Acute stress reaction |
|  | F43.1 – Post-traumatic stress disorder |
|  | F43.2 – Adjustment disorders |
|  | F43.8 – Other reactions to severe stress |
|  | F43.9 – Reactions to severe stress, unspecified |
| **Self-Harm/Suicidal Ideation** | |
| Self-harm | ^X60-69, ^X70-79, ^X80, ^X81, ^X82, ^X83, ^X84, ^T14.91, ^Y87.0, |
| Suicidal ideation | ^R45.81, ^R45.851 |

*Note.* ICD-10-AM = International Classification of Diseases 10th Edition – Australian Modification; CoDDaC = Controlled Drugs Data Collection.

**Supplementary Table S3.** Mean social-emotional competency and difficulty scores for the 5-profile solution.

| **Girls** | | | | | | | | | | | |
| --- | --- | --- | --- | --- | --- | --- | --- | --- | --- | --- | --- |
|  | Social-Emotional Competencies | | | | | Psychopathology | | | | | |
| Profiles | Self-Awareness | Self-Management | Social Awareness | Relationship Skills | Responsible Decision-Making | | Emotional Symptoms | Peer Relationship Problems | Conduct Problems | Hyperactivity-Inattention | |
| Low Mental Health | -0.364 | -0.963 | -0.576 | -0.576 | -0.465 | | 0.557 | 0.531 | 0.805 | 0.673 | |
| Externalising symptoms with poor Self-Management | -0.144 | -0.570 | -0.253 | -0.081 | -0.273 | | 0.237 | 0.058 | 0.413 | 0.433 | |
| Internalising symptoms with poor Relationship Skills | -0.171 | -0.008 | -0.125 | -0.534 | -0.021 | | 0.433 | 0.580 | 0.129 | 0.123 | |
| Average Mental Health | -0.131 | 0.058 | -0.064 | -0.045 | --0.158 | | 0.021 | -0.053 | -0.028 | 0.182 | |
| Complete Mental Health | 0.207 | 0.228 | 0.205 | 0.231 | 0.241 | | -0.233 | -0.161 | -0.213 | -0.346 | |
| **Boys** | | | | | | | | | | | |
|  | Social-Emotional Competencies | | | | | Psychopathology | | | | |  |
| Profiles | Self-Awareness | Self-Management | Social Awareness | Relationship Skills | Responsible Decision-Making | | Emotional Symptoms | Peer Relationship Problems | Conduct Problems | Hyperactivity-Inattention | |
| Low Mental Health | -0.347 | -0.807 | -0.563 | -0.452 | -0.466 | | 0.289 | 0.272 | 0.656 | 0.571 | |
| Externalising symptoms with poor Self-Management | -0.015 | -0.678 | 0.050 | 0.173 | 0.052 | | 0.327 | 0.074 | 0.385 | 0.284 | |
| Internalising symptoms with poor Relationship Skills | -0.139 | -0.030 | -0.137 | -0.602 | -0.022 | | 0.457 | 0.575 | 0.142 | 0.157 | |
| Average Mental Health | -0.128 | 0.045 | -0.148 | -0.105 | -0.179 | | -0.038 | -0.038 | -0.010 | 0.155 | |
| Complete Mental Health | 0.211 | 0.311 | 0.263 | 0.261 | 0.243 | | -0.194 | -0.156 | -0.258 | -0.347 | |
